# Depth distributions of bacteria for the *Pseudomonas aeruginosa*-infected burn wounds treated by methylene blue-mediated photodynamic therapy in rats: effects of additives to photosensitizer

**DOI:** 10.1117/1.JBO.27.1.018001

**Published:** 2022-01-27

**Authors:** Roma R. Sarker, Yasuyuki Tsunoi, Yasue Haruyama, Shunichi Sato, Izumi Nishidate

**Affiliations:** a Tokyo University of Agriculture and Technology, Graduate School of Bio-Applications and Systems Engineering, Koganei, Japan; b Bangladesh Agricultural University, Department of Medicine, Faculty of Veterinary Science, Mymensingh, Bangladesh; c National Defense Medical College Research Institute, Division of Bioinformation and Therapeutic Systems, Tokorozawa, Japan

**Keywords:** *Pseudomonas aeruginosa*, antimicrobial photodynamic therapy, LED, methylene blue, depth distribution of bacteria, burn wound

## Abstract

**Significance:**

*Pseudomonas* *(P.) aeruginosa*, a common cause of infection in burns, acquires antibiotic resistance easily and forms biofilms efficiently. Thus, it is difficult to control *P. aeruginosa* infection in burn wounds, which causes lethal septicemia. Antimicrobial photodynamic therapy (aPDT) is attractive as a new strategy to treat burn wound infections with drug-resistant bacteria.

**Aim:**

We examined the efficacy of methylene blue (MB)-mediated aPDT with various additives in a tissue depth-resolved manner to find conditions that minimize the bacterial invasion.

**Approach:**

We applied MB-mediated aPDT with LED array illumination to an extensive, full-thickness burn infected with *P. aeruginosa* in rats for three consecutive days (days 0, 1, and 2). On day 2, the depth distributions of bacteria were assessed based on the histological analysis using Gram staining. We examined how the addition of ethylenediaminetetraacetic acid (EDTA), ethanol, and dimethyl sulfoxide (DMSO) affected the efficacy of aPDT.

**Results:**

Pure MB-mediated aPDT significantly reduced the numbers of bacteria with biofilms on the wound surface and in the epidermis compared with those for the control tissue (saline only). However, there were many bacteria in the deeper region of the tissue. In contrast, MB/EDTA/ethanol/DMSO-mediated aPDT minimized the numbers of bacteria in the broad depth region of the tissue. Still, a limited number of bacteria was observed in the subcutaneous tissue.

**Conclusions:**

The depthwise analysis of bacteria demonstrated the efficacy of the MB-mediated aPDT with the addition of EDTA, ethanol, and DMSO in controlling burn wound infections. However, further improvement of the therapy is needed to suppress bacterial migration into the deep tissue completely.

## 1 Introduction

Patients with severe burns have a higher susceptibility to infections due to the destruction of their cutaneous barrier and altered systemic immune responses.^1^ Bacteria can invade the deeper layers of a burned tissue and migrate into the bloodstream, possibly leading to septicemia, the common cause of death of patients with severe burns.^2^ In recent years, drug-resistant bacteria have been increasing owing to the large-scale use of antibiotics.^3^ Methicillin-resistant *Staphylococcus aureus* and multidrug-resistant *Pseudomonas* *(P.) aeruginosa* are common drug-resistant bacteria in burn wounds; they efficiently form biofilms that provide robust protection. No effective methods have been established to treat such burn wounds infected with drug-resistant bacteria.

Antimicrobial photodynamic therapy (aPDT) is an attractive treatment strategy for burn wound infections with drug-resistant bacteria.^4^ In aPDT, a photosensitizer (PS) is excited by light, and the excited energy is transferred to the oxygen in tissues, producing reactive oxygen species (ROS), such as singlet oxygen. ROS can kill a wide spectrum of bacteria, including drug-resistant bacteria, through direct oxidation. We previously found that methylene blue (MB)-mediated aPDT with the addition of ethylenediaminetetraacetic acid (EDTA), ethanol, and dimethyl sulfoxide (DMSO) was effective for controlling *P. aeruginosa* infections in a rat extended, full-thickness burn model.^5^ EDTA, ethanol, and DMSO play a role in suppressing biofilm formation, increasing singlet oxygen productivity, and enhancing drug delivery into the tissue, respectively.^6^^–^^9^ Still, we observed rapid bacterial regrowth on the wound surface even after a daily application of aPDT for a week, suggesting a powerful invasion of residual bacteria into the deeper tissue. Thus, it is necessary to examine the efficacy of aPDT in a tissue depth-resolved manner to improve the treatment. To the best of knowledge, there are no reports on the depth-resolved analysis of the aPDT efficacy in tissues.

In this study, we applied MB-mediated aPDT to extensive, full-thickness burns infected with *P. aeruginosa* in rats for three consecutive days (days 0, 1, and 2). On day 2, tissue biopsy was performed, and the depth distributions of bacteria were assessed based on the histological analysis using Gram staining. We previously reported preliminary results with the limited conditions and limited number of animals.^10^ In this study, we compared the results in the rats (n=4) receiving five different treatments: control (treatment with only saline); the PS mixture only (MB/EDTA/ethanol/DMSO without light); aPDT with only MB; aPDT with MB/EDTA/ethanol (without DMSO); and aPDT with MB/EDTA/ethanol/DMSO.

## 2 Materials and Methods

### 2.1 Bacterial Strain and Culture

We used a strain of *P. aeruginosa*, ATCC 27853 (Microbiologics, Inc., Minnesota), which was reported to be resistant to several antibiotics, including penicillin G, kanamycin, and vancomycin.^11^ A stock bacterial suspension was maintained by growing the bacteria in the brain heart infusion broth (05508, Nissui Pharmaceutical Co., Tokyo, Japan). A suspension of *P. aeruginosa* was cultured in a static incubator for 18 h and a 37°C shaking incubator at 140  strokes/min for 6 h. The bacteria were then centrifuged at 3000 rpm for 20 min, resuspended in phosphate buffer saline (PBS) at a density of $3\times 10^{9}\;\mathrm{CFU}/\mathrm{ml}$, and stored in a deep freezer at −80°C before use.

### 2.2 Photosensitizer and Additives

A hydrophilic cationic PS, MB (319112; Sigma Aldrich, St. Louis, Missouri), was used at a concentration of 965  μM in saline. Hydrophilic cationic PSs can bind with negatively charged lipopolysaccharide molecules in the outer membrane of Gram-negative bacteria.^12^ To the MB solution, 10-mM EDTA disodium dihydrate [2NA(EDTA.2Na)] (345-01865; Dojindo Laboratories Co., Kumamoto, Japan), 10% ethanol (057-00451; Fujifilm Wako Pure Chemical Co., Osaka, Japan), and 25% DMSO (043-07216; Fujifilm Wako Pure Chemical Co., Osaka, Japan) were added. The concentrations of ethanol, at 10%, and EDTA, at 10 mM, were based on the results of our previous *in vitro* study on the aPDT against *P. aeruginosa* in biofilms.^13^ Meanwhile, the concentration of DMSO was determined based on the observation that 25% DMSO displayed no dark toxicity on the bacteria in the biofilm *in vitro* (data not shown). Here, we compared the efficacies of aPDT with only MB; aPDT with MB, EDTA, and ethanol (MB/EDTA/ethanol); and aPDT with MB, EDTA, ethanol, and DMSO (MB/EDTA/ethanol/DMSO). In addition, we assessed the efficacies for the rats treated with only saline (control) and those treated with the MB/EDTA/ethanol/DMSO solution in the dark to assess the dark toxicity of the PS mixture, and the results were compared with those for the above-described three aPDT groups.

### 2.3 Light Source

**Fig. 1:**
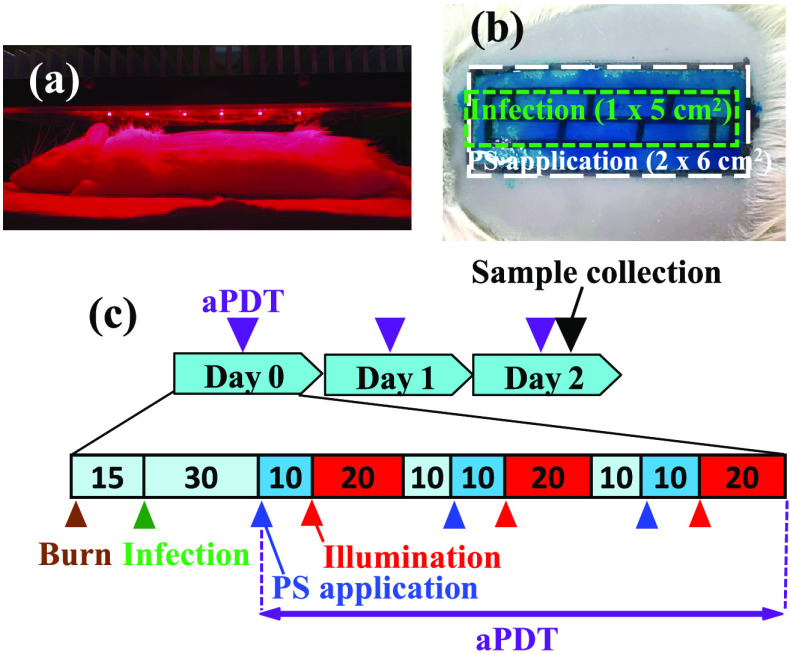
Setup and timeline of the *in vivo* aPDT experiment. (a) A rat under LED illumination. (b) The regions for PS mixture applications ($2\times 6\;{\mathrm{cm}}^{2}$) and infections ($1\times 5\;{\mathrm{cm}}^{2}$) on the burn wound ($4\times 10\;{\mathrm{cm}}^{2}$). (c) The timeline of the experiment.

A 3×6 array of LEDs with a center wavelength of 665 nm and a spectral width of 17.2 nm in FWHM (Effect Corporation, Tokyo, Japan) was used as the light source for aPDT [Fig. 1(a)]. The area of burned skin ($2\times 6\;{\mathrm{cm}}^{2}$), on which the PS mixture solution was applied, was illuminated at a distance of 2.5 cm from the surface of the glass plate covering the LEDs. The average light intensity, which was measured using a powermeter (Vega P/N, 7Z01560; Ophir Optronics Solutions Ltd., Jerusalem, Israel), was $45\;\mathrm{mW}/{\mathrm{cm}}^{2}$ at the wound surface. The intensity distribution on the wound surface was highly uniform with a variation smaller than 5%.

### 2.4 Establishment of Burn Wound Infection

All animal procedures were approved by the Ethics Committee of Animal Care and Experimentation in the National Defense Medical College, Japan (permission number 17020). Male Sprague–Dawley rats (7 to 8 weeks old and 210 to 280 g) (Japan SLC, Inc., Shizuoka, Japan) were used. First, the rats were anesthetized by the intraperitoneal injection of 50  mg/kg pentobarbital. Then, their dorsal hair was clipped with a hair clipper and shaved with an electric shaver and washed. Before a burn was induced, 0.01  mg/kg buprenorphine was intramuscularly injected into the rat thigh muscle for pain relief. A deep burn of ∼20% of the total body surface area ($4\times 10\;{\mathrm{cm}}^{2}$) was induced by exposing the dorsal skin of the rat to 98°C heated water for 10 s through a Walker–Mason template.^14^ Immediately after the burns were induced, the rats were resuscitated with an intraperitoneal injection of saline solution at 25  mg/kg. At 15 min postburn, the infection was established by applying a 100-μl suspension of *P. aeruginosa* at $10^{8}\;\mathrm{CFU}/\mathrm{ml}$ onto the central area ($1\times 5\;{\mathrm{cm}}^{2}$) of the burn [Fig. 1(b)].

### 2.5 Grouping of Animals, aPDT, and Humane Endpoint

The rats with burn wound infection were divided into five treatment groups with four (n=4 for each group): (1) only saline (control), (2) a PS mixture (MB/ethanol/EDTA/DMSO) in the dark (PS-only), (3) aPDT with only MB (aPDT I); (4) aPDT with MB/ethanol/EDTA (aPDT II), and (5) aPDT with MB/ethanol/EDTA/DMSO (aPDT III).

Figure 1(c) shows the timeline chart of the experimental procedures for the aPDT I to III groups. At 30 min after infection, a 125-μl PS mixture (only MB, MB/ethanol/EDTA, or MB/ethanol/EDTA/DMSO) was applied onto an area of $2\times 6\;{\mathrm{cm}}^{2}$ at the center of the burn, including the infected area [Fig. 1(b)]. Ten minutes later, the burn area was illuminated with the LED light for 20 min. The PS mixture application and illumination were consecutively repeated three times as one daily treatment. For the control rats, saline was applied, whereas for the PS-only rats, the PS mixture (MB/ethanol/EDTA/DMSO) was applied; these groups had the same timeline as the three aPDT groups but received no illumination. The treatment was conducted every 24 h for 3 days (days 0, 1, and 2). After the daily treatment, the wound surface was covered with a moist wound dressing sheet (Zuiko Medical Corp., Osaka, Japan) and wrapped in bandages (Daiei Co., Ltd, Osaka, Japan) until the next day’s treatment for all groups of rats. Meanwhile, all of the rats were kept one per cage and allowed easy access to water and food. Finally, the humane endpoint was set to the loss of more than 25% of the body weight on day 0.

### 2.6 Sample Collections

Before and after each day’s treatment, swabbing of two randomly chosen regions of interest (ROIs) on the infected wound surface [Fig. 1(b)] was done with two sterile saline-wetted cotton swabs to evaluate the CFUs on the wound surface. Immediately after the treatment on day 2, two biopsy samples that included all skin layers and subcutaneous tissue, were randomly collected from the infected area ($1\times 5\;{\mathrm{cm}}^{2}$) on each rat using an 8 mm in diameter biopsy punch. The biopsy samples were used for histological analysis to assess bacterial depth distributions.

### 2.7 Colony-Forming Assays

The CFUs on the wound surface were assessed by obtaining two cotton swabs, one taken from one ROI and the other taken from a different ROI, and inserting them separately into 1 ml of sterile saline and vortexed to detach the bacteria. The halves of those suspensions, at 500  μl each, were mixed together to make a 1-ml sample. Each surface swabbing sample was serially diluted with saline and spread onto nutrient agar media (05514; Nissui Pharmaceutical Co., Tokyo, Japan) in a dish. Since we previously observed that for the same *P. aeruginosa* strain, the nutrient agar media showed a higher detectability than the selective media [nalidixic acid-cetrimide (NAC) agar], we used nutrient agar media in this study. When bacteria other than *P. aeruginosa* were involved in the samples, we could distinguish them on the basis of the morphologies, sizes, and colors of their colonies. In such cases, we subcultured those colonies on an NAC agar plate for identification. We excluded those bacteria that were confirmed not to be *P. aeruginosa* from evaluation. However, we seldom observed other types of bacteria, probably indicating a clean experimental environment. After incubating the dishes at 37°C for 18 h, the colonies on the media surface were counted to obtain the CFU/ml values.

### 2.8 Histological Analysis of Bacterial Depth Distributions

The depth distributions of the bacteria in the burned tissues were determined with Gram staining, a fast and effective and standard staining technique for examining clinical biopsy samples. Gram staining technique can identify both large aggregates of clustered bacteria and individual detached bacteria in biofilms.^15^ One problem of Gram staining is the relatively low color contrast between bacteria and their background tissue. We expected that fluorescence-based detection techniques, such as immunofluorescence imaging^16^ and live imaging using GFP-tagged *P. aeruginosa*,^17^ enabled higher-contrast detection. However, our preliminary data showed that autofluorescence considerably disturbed the detection of the GFP fluorescence signals. We also tested the use of CTC (5-cyano-2,3-ditolyl tetrazolium chloride) and CFDA (5(6)-carboxyfluorescein diacetate) to detect bacteria in the tissues, but similar problems (disturbance by autofluorescence) arose. Therefore, we chose the Gram-staining technique. It should be noted that both alive and dead bacteria could be detected by this method.

The biopsied samples obtained from the wound of each rat were fixed in 4% paraformaldehyde in a PBS (163-20145; Fujifilm Wako Pure Chemical Co., Osaka, Japan) overnight and preserved in 70% ethanol until paraffin embedding. Then, the specimens were processed in Tissue-Tek VIP^®^ 6 (VIP 6-J0; Sakura Finetek Japan Co., Ltd., Tokyo, Japan) within a week, embedded in paraffin using a Tissue-Tek TEC Plus Dispensing Console (TEC-P-DC-J0; Sakura Finetek Japan Co., Ltd., Tokyo, Japan), sliced into 7-μm sections using a sliding microtome (TTM-200; Sakura Finetek Japan Co., Ltd., Tokyo, Japan), and placed on glass slides (MAS-02; Matsunami Glass Ind. Ltd., Tokyo, Japan).

The sections were Gram-stained with Gram–Hucker’s Stain solution I (4116-2), II (4117-2), and III (4118-2) (Muto Pure Chemicals Co., Ltd., Tokyo, Japan) after de-paraffinization with xylene and a decreasing ethanol gradient. Next, an increasing ethanol gradient and xylene were used to dehydrate the sections. The specimens were then covered with a cover glass and observed under a Carl Zeiss Inverted Microscope (Axiovert 200; Carl Zeiss, Göttingen, Germany) at 1000× magnification to assess the depth distributions of the bacteria. For each Gram-stained section, four vertical regions with a width of 600  μm and a minimum horizontal separation of 700  μm were analyzed. In each vertical region, a narrower vertical ROI with a width of 120  μm was randomly chosen, and the number of bacteria was counted for every 100  μm depth section from the region on the skin surface down to the muscle layer. For cluster forming bacteria, we estimated the approximate numbers. When the total numbers of bacteria for each depth section were exceeded, e.g., 200 and 300, we scored the numbers as “>200,” “>300,” respectively. As described above, two biopsied samples were obtained from each rat, and bacterial counting was conducted for eight ROIs in each rat. Thus, the total number of ROIs was 32 for each group of rats (n=4), and the averaged numbers for 32 ROIs were used to compare the groups.

### 2.9 Statistical Analysis

Statistical analysis of the data was performed using GraphPad Prism 6 software. Nonparametric two-way ANOVA was used to compare the average numbers of bacteria for each depth section between the groups. Statistical significance was determined for the results of each group in comparison with those of the control group or those of the aPDT I group. A difference with a P-value<0.05 was considered statistically significant.

## 3 Results

### 3.1 Bacterial Numbers on the Wound Surface

**Fig. 2:**
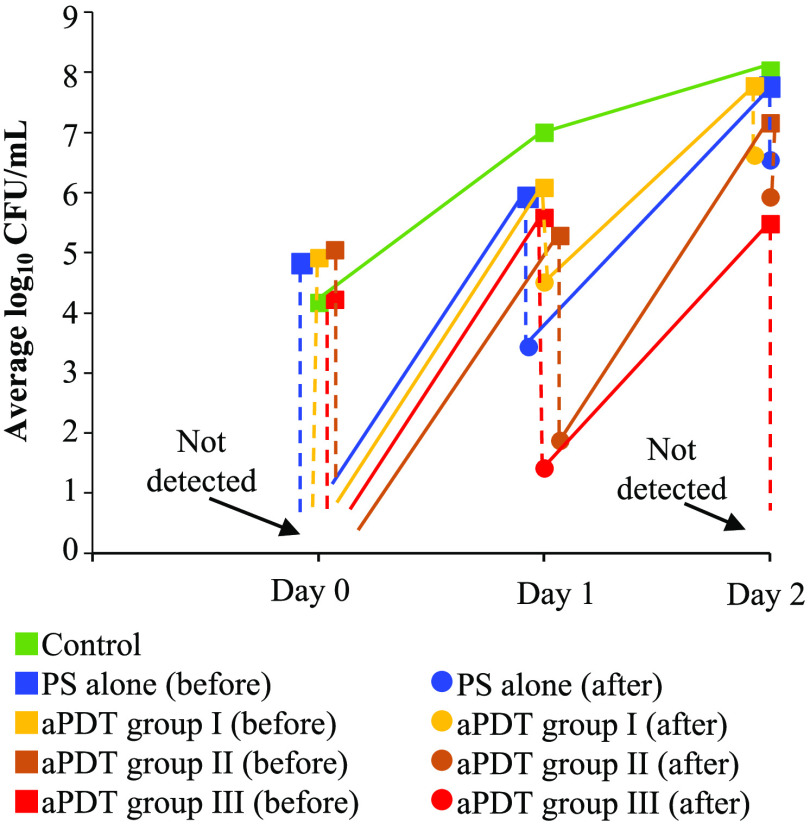
Time courses of bacterial numbers on the wound surface in different groups of rats (n=4 in each group). On each day, both of the numbers before (squares) and after (circles) treatment are shown except for the control; the corresponding two plots are connected with a vertical dashed line.

No rats reached the defined humane endpoint in this study. Figure 2 shows the time courses of the average numbers of CFUs on the wound surfaces, which were evaluated for swabbing samples, in all groups of rats. Before the treatment on day 0, the average CFUs were similar for all groups at around $4.6\;\log_{10}\;\mathrm{CFU}/\mathrm{ml}$. After the treatments, no bacteria were detected in any treatment groups, including the PS-only group, indicating the efficacy of aPDT with any mixtures as well as the dark toxicity of the PS mixture. However, the bacteria on the wound surfaces were rapidly regrown by the timepoint before the treatment on day 1 for all treatment groups, although their numbers were smaller than that of the control group by 0.9 to $1.7\;\log_{10}$. The bactericidal effects of the treatments on day 1 were different depending on the groups; CFU reductions were $1.6\;\log_{10}$ for aPDT I (MB only), $3.4\;\log_{10}$ for aPDT II (MB/ethanol/EDTA), and $4.2\;\log_{10}$ for aPDT III (MB/ethanol/EDTA/DMSO). The efficacy of aPDT with only MB was lower than that of the treatment with only the PS mixture, i.e., the dark toxicity. After the treatments on day 1, efficient bacterial regrowth occurred again in all treatment groups. At the timepoint before the treatment on day 2, the bacterial numbers for the PS-only, aPDT I, and aPDT II groups were similar to that of the control group, showing monotonical increases in the number of bacteria at the timepoints before the treatment of each day during days 0 to 2. On the other hand, the bacterial number for the aPDT III group was slightly smaller than that at the timepoint before the treatment on day 1. For aPDT group III, the treatment on day 2 greatly reduced the number of bacteria ($>5.3\text{-}\log_{10}$ reduction), resulting in no bacterial detection on the wound surface.

For other treatment groups, on the other hand, bactericidal effects of the treatments on day 2 were limited to ∼1.2-log reduction, indicating time-dependent decreases in the efficacy. These results showed the importance of adding DMSO to the PS mixture in controlling the numbers of bacteria on the wound surfaces.

### 3.2 Bacterial Depth Distributions

**Fig. 3:**
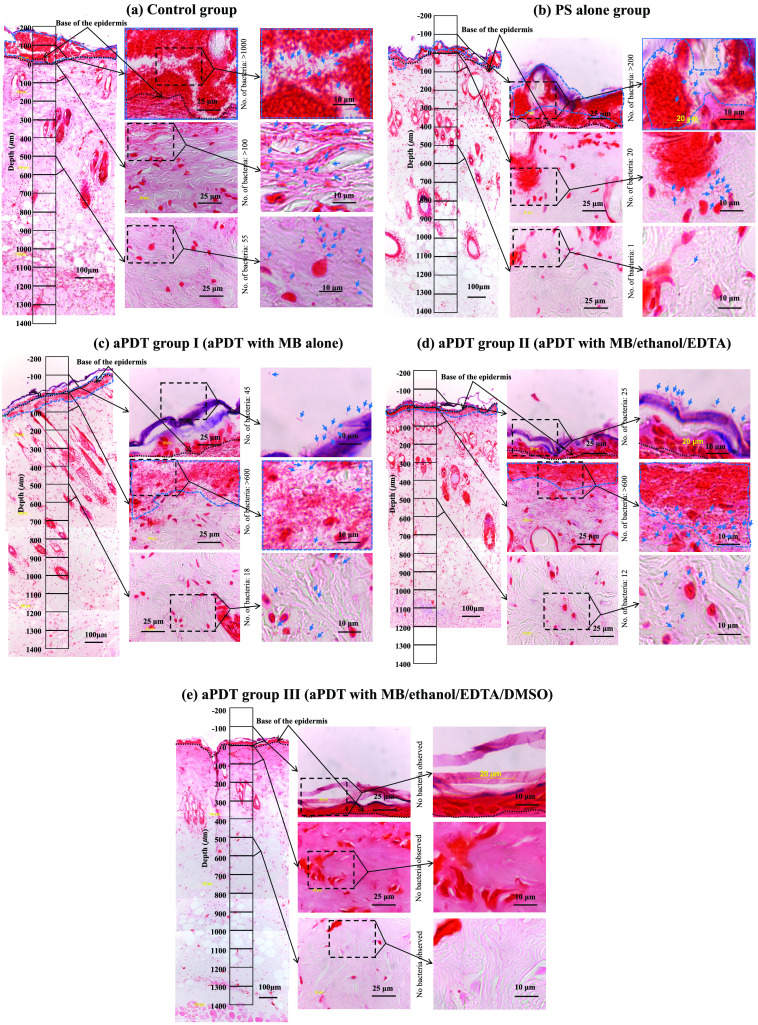
Representative Gram-stained images of *P. aeruginosa*-infected burned skin sections of rats after five different treatments on day 2: (a) saline only (control), (b) MB/ethanol/EDTA/DMSO in the dark (PS-only), (c) aPDT with only MB (aPDT I), (d) aPDT with MB/ethanol/EDTA (aPDT II), and (e) aPDT with MB/ethanol/EDTA/DMSO (aPDT III). Three columns of images were taken at different magnifications for each group. The magnification of the images in the first column is 200×. A depth scale with box segments with a depth of 100  μm and a width of 120  μm is displayed, and the depth origin (0  μm) is set at the base of the epidermis (black dotted line). The negative depths (0 to −200  μm) represent the epidermis, including the stratum corneum and a region above it. The regions of 0 to ∼600  μm and ∼600 to ∼1400  μm correspond to the dermis and hypodermis, respectively. Higher magnification images of three representative segments, the epidermis (−100 to 0  μm), the uppermost region of the dermis (0 to 100  μm), and the deepest region of the dermis (500 to 600  μm) are shown in the second column. The magnified images of the black dashed line boxes in the second column are shown in the third column with the indicated bacteria (blue arrows). The regions defined with blue dashed lines indicate clustered bacteria with or without biofilms.

Figure 3 shows the representative images of the Gram-stained sections of the wounds after the treatment on day 2 for all groups, with three columns of images taken at different magnifications for each group. The magnification of the images in the first column image was 200×. A depth scale with box segments with a depth of 100  μm and a depth of 120  μm is displayed, and the depth origin (0  μm) is set at the base of the epidermis (black dotted line). The negative depths (0 to −200  μm) represent epidermis, including the stratum corneum and a region above this. The depth regions of 0 to ∼600  μm and ∼600 to ∼1400  μm correspond to dermis and hypodermis, respectively. Higher magnification images of three representative segments: the epidermis (−100 to 0  μm), the uppermost region of the dermis (0 to 100  μm), and the deepest region of the dermis (500 to 600  μm) are shown in the second column. Further magnified images of black dashed line boxes in the second column images are shown in the third column, in which bacteria are indicated with blue arrows. Regions defined with blue dashed lines indicate clustered bacteria with/without biofilms.

In the wounds of the control rats [Fig. 3(a)], massive high-density clustered bacteria with biofilms were seen in and on the epidermis (first column). There were also many bacteria in the uppermost dermal region (middle, third column), and the deepest dermal region (bottom, third column). In the wounds of the PS-only group rats [Fig. 3(b)], high-density clustered bacteria with biofilms were observed in or on the epidermis; however, their areas were smaller than those in the control group. There were many clustered bacteria in the uppermost dermal region (middle, third column). Unexpectedly, only a limited number of bacteria was observed in the deepest dermal region (bottom, third column). Meanwhile, the wounds of the rats with aPDT I (MB only) and aPDT II (MB/ethanol/EDTA) exhibited similar distributions of bacteria [Figs. 3(c) and 3(d)]. Neither high-density clustered bacteria nor biofilms were observed in or on the epidermis. However, there were high-density clustered bacteria, probably with biofilms, in the uppermost dermal regions [middle, third column; Figs. 3(c) and 3(d)]. Some bacteria were seen in the deepest dermal regions, but their numbers appeared to be smaller than those in the control group [bottom, third column; Figs. 3(c) and 3(d)]. In the wounds of the rats with aPDT III (MB/ethanol/EDTA/DMSO), neither bacteria nor biofilms were found clearly in any regions [Fig. 3(e)].

**Fig. 4:**
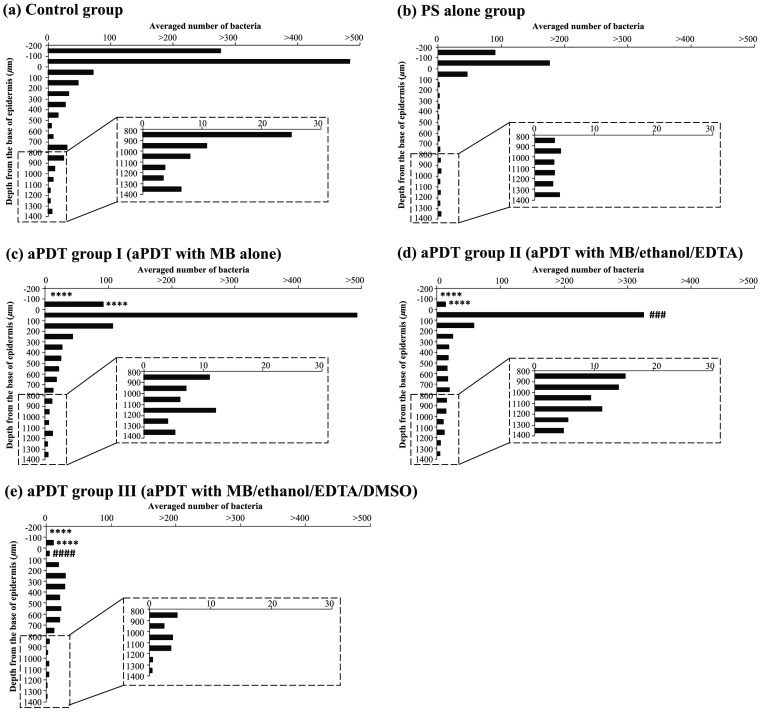
Depth distributions of *P. aeruginosa* in the burned skins of rats that were treated under five different treatment conditions: (a) saline only (control), (b) MB/ethanol/EDTA/DMSO in the dark (PS-only), (c) aPDT with only MB (aPDT I), (d) aPDT with MB/ethanol/EDTA (aPDT II), and (e) aPDT with MB/ethanol/EDTA/DMSO (aPDT III). The depth at 0  μm indicates the base of the epidermis. The numbers of bacteria were counted for every 100-μm depth section with the width of 120  μm from the skin surface down to the muscle layer based on the Gram-stained tissue sections. The values indicate the average numbers of the bacteria for 32 ROIs in total (four rats, two sections per rat, four ROIs per section). ****P<0.0001, compared with the bacterial number in the corresponding region of the control group. ^####^P<0.0001 and ^###^P<0.001, compared with the bacterial number in the corresponding region in the aPDT I group.

Figure 4 shows the average depth distributions of bacteria (n=4 for each group) after the treatment on day 2, with the bacterial distributions in the region deeper than 800  μm shown at a higher magnification. In the control group [Fig. 4(a)], there were large numbers of bacteria in and on the epidermis, with ∼480 in the segment of 0 to −100  μm (the epidermis) and ∼280 in the segment of −100 to −200  μm (the region above the stratum corneum). Meanwhile, the treatment with only the PS mixture (in the dark) considerably reduced the numbers of bacteria in the epidermis and the region above the stratum corneum to ∼180 and ∼90, respectively; however, there were no significant differences in the numbers between the control and PS mixture groups. In contrast, the numbers of bacteria in the epidermis and the region above the stratum corneum were significantly decreased by aPDT of any type. The bacterial count was decreased to ∼90 and 0, respectively, in the aPDT I group [Fig. 4(c), P<0.0001]; to ∼10 and 0, respectively, in the aPDT II group [Fig. 4(d), P<0.0001]; and to ∼10 and 0, respectively, in the aPDT III group [Fig. 4(e), P<0.0001], respectively, compared with those in the control group. However, in the wounds of the aPDT I group [Fig. 4(c)], there were many bacteria, ∼490, in the segment of 0 to 100  μm (the uppermost region of the dermis). On the other hand, the number of bacteria in this segment was significantly smaller in the wounds of the aPDT II group, at ∼330 [Fig. 4(d); P<0.001] and the aPDT III group, at ∼10 [Fig. 4(e); P<0.0001], than those in the aPDT I group.

For the region deeper than 100  μm, the aPDT I and II groups showed similar bacterial distributions, and the distribution of the aPDT III group was smaller than that of the aPDT I and II groups. Unexpectedly, the PS-only group showed a smaller bacterial distribution than the aPDT I group or aPDT II group. Among all of the groups, the aPDT III group had the smallest bacterial numbers for the deepest region, i.e., the region deeper than 1200  μm in the hypodermis.

## 4 Discussion

We previously showed that MB-mediated aPDT effectively killed *P. aeruginosa* in mature biofilms when ethanol and EDTA were added to the PS mixture *in vitro*.^13^ In addition, MB-mediated aPDT with the addition of ethanol, EDTA, and DMSO for 7 consecutive days suppressed bacterial invasion from the wound to the inside of the rats with a *P. aeruginosa*-infected, extended, full-thickness burn.^5^ However, some rats under this treatment showed certain bacterial invasion, and the rescue rate was limited to 78.6% (11 out of 14 rats). Thus, in this study, we aimed to improve the efficacy of aPDT by evaluating the bacterial depth distributions in the tissues under the different treatment conditions using the same model.

At the timepoint before the treatment on day 2, the bacterial numbers for the PS-only, aPDT I, and aPDT II groups were similar to that of the control group, showing monotonical increases in the number of bacteria at the timepoints before the treatment of each day during days 0 to 2. On the other hand, bacterial number for the aPDT III group was slightly smaller than that at the timepoint before the treatment on day 1. For aPDT group III, the treatment on day 2 greatly reduced the number of bacteria ($>5.3\text{-}\log_{10}$ reduction), resulting in no bacterial detection on the wound surface.

For other treatment groups, on the other hand, bactericidal effects of the treatments on day 2 were limited to ∼1.2-log reduction, indicating time-dependent decreases in the efficacy. These results showed the importance of adding DMSO to the PS mixture in controlling the numbers of bacteria on the wound surfaces. The time-dependent decrease in the efficacy observed in the aPDT I and II groups are attributable to insufficient bactericidal effects, leaving active bacteria in the subsurface tissue after the treatment [Figs. 3(c) and 3(d)]. Those bacteria can efficiently regrow and produce biofilms which protect themselves in a time-dependent manner, resulting in the time-dependent decrease in the aPDT efficacy. However, bacterial adaptation to aPDT, which is acquired in the tissue region with nonlethal doses, can also affect the time-dependent efficacy of the treatment.^18^ We will address this issue in a future study.

Since the wound surfaces under the different treatment conditions showed considerable differences in bacterial numbers on day 2 (Fig. 2), we focused on the bacterial depth distributions at this timepoint. Massive, dense bacterial clusters with biofilms were observed in the regions above the epidermal base in the control group [Figs. 3(a) and 4(a)]; these clusters were significantly suppressed by MB-mediated aPDT [the aPDT I group, Figs. 3(c) and 4(c)] and MB-mediated aPDT with the ethanol and EDTA [the aPDT II group, Figs. 3(d) and 4(d)]. However, the wounds in the aPDT I and II groups still had large numbers of bacteria with biofilms in the top layer of the dermis [Figs. 3(c) and 3(d)], indicating limited treatment depths. The addition of DMSO to the PS mixture (aPDT III) drastically improved aPDT’s efficacy to minimize the numbers of bacteria not only in the top layer of the dermis but also in the deepest regions at more than 1200  μm [Fig. 3(e) and 4(e)], likely due to the enhanced penetration of all of the molecules in the PS mixture by DMSO, thus improving the antibacterial and antibiofilm efficacy of aPDT in the deeper regions. In addition to enhancing drug penetration, DMSO has bactericidal activities against *P. aeruginosa*, inhibiting the production of virulence factors and preventing the formation of *P. aeruginosa* biofilms.^19^^–^^21^ The blood vessels in the deepest regions should be viable even under a full-thickness burn and still be able to carry invaded bacteria through the blood flow. Thus, reducing the bacteria in these regions is particularly important to control bacterial invasion to inside the body.

Unexpectedly, the application of only the PS mixture (without light) was also highly effective in reducing the number of bacteria in the regions at all depths [Fig. 4(b)] when compared with the control [Fig. 4(a)], indicating the robust dark toxicity of the PS mixture. Furthermore, the numbers of bacteria in the depth regions from 100 to 900  μm in the dermis and hypodermis were lowest even when compared with those in the corresponding depth regions in any aPDT group [Figs. 4(c)–4(e)], likely because neither photobleaching nor oxygen consumption occurred in the PS-only group, leading to efficient bacterial killing by the dark toxicity of the PS in the broad depth region. MB is known to be metabolized to yield demethylated products, typically azure B, which is more cytotoxic than MB.^22^ Those products can be associated with the dark toxicity observed in this study. To fully understand the mechanism of the dark toxicity, we need to analyze the metabolism of MB in the infected, burned tissue in the future, for which liquid chromatography of samples extracted from the tissue will be useful.

In aPDT, the photobleaching of a PS and oxygen deletion can limit the treatment’s efficacy. In our previous study, we investigated the toxicity of the same PS mixture on the normal rat skin *in vivo* by assessing the mitochondrial enzyme activity with nicotinamide adenine dinucleotide-diaphorase (NADH-D) staining. The results showed that applying the PS mixture to the normal skin for 7 consecutive days did not reduce the cell viability of the skin,^5^ suggesting the use of the PS mixture as an antibacterial agent to control wound infection without light. However, we observed that the bactericidal effect of the PS mixture by itself was reduced day by day, and the application of the PS mixture for 7 consecutive days could not rescue infected burned rats, likely due to the efficient formation of biofilms on the wound surface that robustly protected bacteria from exposure to the PS mixture [Fig. 3(b)]. Thus, an aPDT with light illumination can be effective for controlling wound infections.

Notably, even under the best aPDT conditions in this study (aPDT III), bacteria were observed in the deepest regions at 800 to 1400  μm and could cause blood-borne bacterial invasion, consistent with our previous observation that all of the rats with burn infections could not be rescued by aPDT.^5^ Since the simultaneous existence of a PS, light, and oxygen is required for the action of aPDT in principle, the depth distribution of each component must be optimized for the further improvement of the efficacy of aPDT. The bacterial distribution in all of the depth regions in the PS-only group being lower than that of the control group [Figs. 4(a) and 4(b)] indicated that molecules of the PS mixture penetrated the deepest region at ∼1400  μm, likely attributable to a broken barrier function of the skin due to the burn. However, optical penetration depth might be decreased due to the relatively high concentration of MB, at 965  μM, used. Thus, optimizing the MB concentration in terms of the optical penetration depth may effectively enhance the efficacy of aPDT.

Finally, since oxygen delivery through blood circulation is blocked in burned tissues, oxygen must be supplied through diffusion to the target tissue from the air or the surrounding uninjured tissue. As described above, oxygen is depleted in the tissue during light illumination in the tissue, and time fractioning of illumination can increase oxygen concentration in the tissue and hence the aPDT efficacy. Even if all of these measures are taken, bacterial invasion cannot be suppressed completely when the rate of bacterial regrowth is very rapid. Therefore, shortening of the treatment interval, 24 h in this study, may be needed in such cases.

## Code, Data, and Materials Availability

The data and images analyzed in this study are included in this article. Additional data and images are available from the corresponding author upon request.
